# Cost-effectiveness analysis of sedation regimens for children undergoing magnetic resonance imaging in Japan: a simulation-based study

**DOI:** 10.1007/s00540-025-03540-8

**Published:** 2025-07-11

**Authors:** Soichiro Obara, Yoshinori Nakata

**Affiliations:** Teikyo University Graduate School of Public Health, 2-11-1 Kaga, Itabashi-ku, Tokyo, 173-8605 Japan

**Keywords:** Cost-effectiveness analysis, Deep sedation, Dexmedetomidine, Propofol, Anesthesiologist

## Abstract

**Purpose:**

This simulation-based cost-effectiveness analysis evaluates various sedation regimens for pediatric magnetic resonance imaging (MRI) in Japan.

**Methods:**

A decision tree model was developed for children aged 3 years with ASA-PS class I/II to compare four sedation regimens: oral triclofos sodium, IV midazolam, IV dexmedetomidine, and IV propofol. The primary outcome was averted sedation failure (aSF). Cost-effectiveness was assessed using the incremental cost-effectiveness ratio (ICER). Deterministic and probabilistic sensitivity analyses, including Monte Carlo simulations and cost-effectiveness acceptability curves (CEACs), were performed. A 0% discount rate was applied. Our systematic literature search determined success rates for each sedation or general anesthesia regimen.

**Results:**

The cost-effectiveness plane demonstrated the efficiency frontier connecting triclofos sodium, propofol, and general anesthesia. Compared to oral triclofos sodium, propofol had an ICER of $3214.06 per additional aSF, which was more favorable than dexmedetomidine (ICER: −$9222.85). Sensitivity analysis showed that ICER values were most sensitive to the success rates of each sedation regimen, followed by the reimbursement rate for anesthesiologist-administered sedation. CEACs confirmed that triclofos sodium and propofol were the most favorable, while midazolam and general anesthesia were less favorable. The probability of cost-effectiveness for propofol varied from 0 to 51.6%, and for triclofos sodium, it ranged from 100 to 38.9%.

**Conclusion:**

Propofol sedation administered by anesthesiologists demonstrated superior cost-effectiveness compared to dexmedetomidine and midazolam sedation administered by non-anesthesiologists, primarily due to higher success rate and lower reimbursement rate for sedation procedures by anesthesiologists. Increasing reimbursement for anesthesiologist-administered sedation may be justifiable, though further real-world validation is needed.

**Supplementary Information:**

The online version contains supplementary material available at 10.1007/s00540-025-03540-8.

## Introduction

The role of anesthesiologists in non-surgical settings has gained increasing importance as the number of procedures performed outside the operating room (OR) continues to rise [1]. Pediatric patients undergoing diagnostic procedures, such as magnetic resonance imaging (MRI), often require sedation to ensure comfort and safety. However, the involvement of anesthesiologists in sedation outside the OR is hindered by barriers, such as reimbursement structures and workforce limitations, in Japan [2]. In Japan, procedural sedation is reimbursed at a lower rate than general anesthesia (GA), regardless of the depth of sedation [2]. This discrepancy discourages anesthesiologists from engaging in procedural sedation, as the financial incentives fail to justify the resources required for specialized care. Given the increasing pressure on the public healthcare system, it is critical to assess the cost-effectiveness of various sedation regimens to optimize resource allocation and ensure sustainability [3].

Health Technology Assessment (HTA) was formally introduced in Japan in 2019, with the aim of managing the rising costs of healthcare and maintaining the sustainability of universal health coverage [3]. The role of HTA in evaluating cost-effectiveness has become increasingly important in the healthcare environment undergoing significant demographic changes, such as an aging population and declining birth rates. Despite the growing importance of cost-effectiveness research, a significant gap remains in the evaluation of anesthesiology practices, particularly in intraoperative and multidisciplinary perioperative interventions [4].

Our previous study assessed the cost-effectiveness of various sedation regimens for pediatric MRI using clinical data from a single children’s hospital [5]. The results suggested that intravenous (IV) sedation administered by anesthesiologists was more cost-effective than oral or rectal sedation, and comparably cost-effective to IV sedation administered by non-anesthesiologists. However, study employed simplified methodologies using data from a single institution in Japan and lacked robustness and generalizability necessary to draw widespread conclusions [5].

The objective of the present study is to evaluate the cost-effectiveness of various sedation regimens for pediatric MRI in Japan. To enhance the robustness and the generalizability of our findings, this study incorporates decision tree modeling with both deterministic and probabilistic sensitivity analyses using data from multiple sources. By adopting a comprehensive health economic analytical method from the viewpoint of the Japanese public healthcare system, this study aims to provide a more rigorous cost-effectiveness evaluation of various sedation regimens for pediatric MRI, offering valuable insights for policymakers and healthcare providers.

## Methods

### Economic analysis

The economic analysis adhered to the Consolidated Health Economic Evaluation Reporting Standards (CHEERS) 2022 guidelines [6] and followed the Guidelines for Economic Evaluation of Healthcare Technologies in Japan [7]. The primary economic outcome was the incremental cost-effectiveness ratio (ICER), which compares the costs of each sedation regimen with its effectiveness in terms of avoiding sedation failure (aSF) (see "Outcome" below). Both deterministic and probabilistic sensitivity analyses were performed to account for uncertainty in the model parameters. The CHEERS 2022 statement checklist is provided as a supplementary material (see Supplementary Materials).

### Study population

The model population was based on the demographic characteristics from our previous study [5]. The patients in the previous study were 3 years of mean age, with 53.4% male and 96.5% classified as ASA-PS class I or II. Seventy-one percent of the population underwent brain MRI. Thus, the target population for this simulation was set as a 3-year-old male patient with ASA-PS class I or II who requires sedation or GA for brain MRI.

The duration of brain MRI in children typically varies. In this study, we used data from a multi-institutional study showing a mean MRI scan time of 38 min (standard deviation: 14 min) [8].

### Setting and location

The cost data used in the analysis were sourced with modeling Japanese healthcare institutions, and the analysis reflects the costs associated with sedation and MRI procedures in the context of the Japanese public healthcare system.

### Comparators

The sedation regimens compared in the analysis were oral triclofos sodium, IV midazolam, IV dexmedetomidine, IV propofol, and GA with sevoflurane inhalation and a supraglottic airway device. These regimens were selected based on data from our previous study [5], and epidemiological data regarding pediatric MRI sedation trends in Japan [9]. Although both triclofos sodium and chloral hydrate have been used in Japan, our decision to include only oral triclofos sodium was based on the national practice patterns. A recent nationwide claims database study showed that oral chloral hydrate was less frequently used than triclofos sodium, and that rectal chloral hydrate was even less common [9]. Therefore, we did not include rectal chloral hydrate in our model to reflect the most prevalent sedation regimens in the real-world practice. IV sedation regimens were modeled with an initial bolus dose, followed by a maintenance infusion. Dexmedetomidine was included due to its increasing use for pediatric procedural sedation outside the OR. In Japan, its use in outpatient settings has been expanding [10]. Internationally, an increasing role of non-anesthesiologists, such as pediatric hospitalists, in administering dexmedetomidine has been reported [11]. Along with efficacy data from a recent RCT [12], these findings support modeling dexmedetomidine administration by non-anesthesiologists. The use of propofol for pediatric procedural sedation by untrained non-anesthesiologists remains a controversial topic [13], and it has not been so common in Japan [5], in alignment with the consensus statement [13]. Therefore, propofol sedation was modeled assuming administration by anesthesiologists, while midazolam and dexmedetomidine were modeled as being administered by non-anesthesiologists, where there are differences in reimbursement rates between sedation administered by anesthesiologists and that by non-anesthesiologists (see "Cost estimation" below).

### Perspective

The analysis was conducted from the perspective of the Japanese public healthcare payer.

### Time horizon

The time horizon for this analysis was limited to a period up to the completion of MRI scan. A 0% discount rate was applied to both costs and outcomes due to the short timeframe, which typically extends only a few days to a few months, even in cases of sedation failure requiring reattempts.

### Model structure

A decision tree (DT) model was developed to simulate the sequential decision-making process for each sedation regimen, with decision points for sedation success or failure. A simplified version of the decision tree model used in this study is illustrated in Supplementary Fig. 1. Detailed descriptions of the model structure, assumptions, and parameters are provided in Supplementary Fig. 2 and its accompanying legend. Each branch of the decision tree corresponds to a clinical action or outcome. All variable names used in the model (including cost and probability parameters) are fully defined in the Supplementary Materials to facilitate reproducibility and clarity of the model structure. A DT model was chosen over a Markov model due to the binary nature of the decisions involved (i.e., sedation success or failure), which does not require the time-dependent state transitions typically associated with Markov models [14].

Based on our previous cost-effectiveness study using clinical data from a single institution in Japan and standard care practices in Japan [5], the following assumptions were made: (1) If oral sedation with triclofos sodium failed, intravenous (IV) sedation with dexmedetomidine was attempted by non-anesthesiologists. (2) If IV sedation with dexmedetomidine or midazolam administered by non-anesthesiologists failed, IV sedation with propofol was attempted by anesthesiologists. (3) If IV sedation with propofol failed, GA with inhalational sevoflurane was provided by anesthesiologists, using a supraglottic airway device (e.g., i-gel). (4) In the case of GA, if the procedure fails, we assume that a second attempt will succeed with 100% probability. This assumption was made to complete the model termination as failure at the GA node would otherwise disrupt the simulation and prevent the completion of the cost-effectiveness analysis.

All the patients undergoing sedation or anesthesia were assumed to be discharged on the same day without significant complications, whether or not the imaging procedures were successful.

### Outcome (effectiveness)

The primary outcome (effectiveness) was averted sedation failure (aSF), calculated as: aSF = 1 − sedation failure rate =  = 1 − (1 − sedation success rate).

"Sedation failure" was defined as the inability to complete the MRI procedure under sedation or anesthesia, requiring either cancelation or cessation of the scan, followed by rescheduling [2]. The success rates for each sedation or GA regimen were derived from a systematic literature search of studies published between 2000 and 2024. The full comprehensive search strategy is provided in Supplemental method (Supplemental Materials). When available, meta-analysis data were used to extract the success rate, which enhanced the reliability of our estimates. For regimens without meta-analysis data, the success rates were calculated from randomized or observational trial data. Detailed search strategy, inclusion and exclusion criteria, data synthesis methods are provided in the Supplementary Materials (see “Supplemental method: Meta-analysis of the success rate for each sedation or general anesthesia regimen”).

### Cost estimation

Major cost components included sedation/anesthesia drugs (oral and IV medications), imaging costs, and procedure costs (Table 1). The largest component of medical costs was staff time, particularly for the physician, surgeon, and anesthesiologist, as determined from the literature [15]. Since the Japanese national medical fee schedule reimburses hospital fees but not individual doctor’s fees [5], staff time costs were estimated based on the associated procedure costs, using the medical remuneration points.

**Table 1:** Key input parameters, ranges in one-way sensitivity analyses, and distributions used in probabilistic sensitivity analyses

Parameter	Unit	Value	Range	Distribution	References
Procedure fees	Unit	Value [$]	Range [$]	Distribution	References
IV sedation by non-anesthesiologists		39.00	–	Gamma	[29]
IV sedation by anesthesiologists		71.50	52.00–195.00	Gamma	[29]
General anesthesia		390.00	–	Gamma	[29]
Peripheral IV infusion		6.83	–	Gamma	[29]
MRI fee		105.30	58.50–105.30	Gamma	[29]
Drug or apparatus costs (#1, #2)	Unit	Value [$]	Range [$]	Distribution	References
Triclofos sodium (oral)	100 mg/ml	0.066	–	Gamma	[30]
Midazolam (IV)	10 mg/2 ml	0.748	0.598–0.748	Gamma	[30]
Dexmedetomidine (IV)	200 µg/2 ml	17.303	7.514–17.303	Gamma	[30]
Propofol (IV)	200 mg/20 ml	4.888	3.861–4.888	Gamma	[30]
Sevoflurane (inh) (#3)	ml	0.00115	–	Gamma	[30]
IV fluid	/500 ml	1.378	–	Gamma	[30]
Supraglottic airway device (i-gel)		4.004	–	Gamma	
Doses of medications	Unit	Value	Range	Distribution	References
Triclofos sodium (oral)	mg/kg	60	30–105	Gamma	[31]
Midazolam (IV)	mg/kg loading, mg/kg/h maintenance	0.2, 0.36	0.1–0.2, 0.18–0.36	Gamma	[32]
Doses of medications	Unit	Value	Range	Distribution	References
Dexmedetomidine (IV)	µg/kg loading, µg/kg/h maintenance	2.8, 1.8	2.3–3.3, 1.4–2.2	Gamma	[33]
Propofol (IV)	mg/kg loading, mg/kg/h maintenance	2.0, 6.0	1.0–2.0, 6.0–12.0	Gamma	[34]
Brain MRI duration	minutes	38.0	24.0–52.0 (mean ± SD)	Normal	[8]
Body weight of 3-year-old-male patient	kg	13.5	11.97–15.03 (mean ± SD)	Normal	[35]
Success rate of sedation (#4)	Provider	Mean	95% CI	Distribution	References
Triclofos sodium (oral)	Non-anesthesiologist	0.940	0.910–0.960	Beta	[26]
Midazolam (IV)	Non-anesthesiologist	0.792	0.206–0.982	Beta	[5, 32, 36]
Dexmedetomidine (IV)	Non-anesthesiologist	0.933	0.946–0.964	Beta	[33]
Propofol (IV)	Anesthesiologist	0.983	0.974–0.993	Beta	[5, 37–46]
General anesthesia	Anesthesiologist	1.000	–	Beta	[5, 36, 47–49]
Pediatric outpatient consultation	Unit	Value [$]	Range	Distribution	References
Pediatric outpatient consultation fee	/patient	46.800	26.650–46.800	Gamma	[26]
Outpatient visits (#5)	/h	2.5	2.5–2.8	Gamma	[50, 51]
Utility					
Averted sedation failure [aSF]	1 − probability of sedation failure		–	–	

(#1) Costs were converted to 2024 U.S. dollars ($) from Japanese Yen (¥) using the exchange rate of ¥1 = $0.0065 as of December 17th, 2024

(#2) Drug costs were based on the Japanese Pharmaceutical Price List (Drug Price List) 2024 [30]. In scenario analyses, drug costs were calculated using the branded drug prices, while in the one-way sensitivity analysis, the range of drug costs was based on the price difference between branded and generic drugs. This approach was used as it is likely more accurate and realistic. However, if no price difference between the generic and branded drugs existed, no range was set

(#3) The consumption rate [mL/h] was calculated using the formula: 3.3 (sevoflurane coefficient) × concentration [%] × gas flow rate [L/min]. For example, the calculation for hourly consumption is as follows: 3.3 × 2.5 [%] × 3 [L/min] = 24.75 [mL]. Therefore, the cost is calculated as: 24.75 [mL] × 0.177 [$/mL] = 4.381 [$]

(#4) To estimate aSF of sedation regimens, success probabilities were derived from multiple published studies. Detailed calculations of the success rate for each sedation regimen are provided in the Supplementary Materials (see "the success rate for each sedation regimen")

(#5) The number of pediatric patients a pediatrician can consult per hour in an outpatient setting was based on data regarding healthcare utilization patterns. In Japan, pediatric patients visit healthcare providers 2.5 times more frequently than in the U.S. [49]. Given that U.S. pediatricians typically see about 1.02–1.12 patients per hour in outpatient settings [50], we assumed that a reasonable estimate for Japan would be 2.8 patients per hour (1.12 × 2.5) on the higher end and 2.5 patients per hour (1.02 × 2.5) on the lower end

*CI* confidence interval, *inh* inhalational, *IV* intravenous, *SD* standard deviation

If MRI was aborted due to sedation failure, additional costs were added: (1) MRI scan: The cost of MRI scan was considered as an opportunity cost, assuming that the aborted scan could be reallocated to another patient for the time frame. (2) Non-anesthesiologist-administered sedation failure: If IV sedation by a non-anesthesiologist (i.e., midazolam or dexmedetomidine) failed, pediatric outpatient consultation fees for non-anesthesiologists were added as an opportunity cost. (3) Anesthesiologist-administered sedation failure: If IV sedation by an anesthesiologist (i.e., propofol) failed, GA fees were added as an opportunity cost, assuming that an anesthesiologist would have provided GA in the OR, had they not been involved in the sedation outside the OR.

### Currency and price year

Currency conversion was performed using an exchange rate of 1 JPY = 0.0065 USD as of December 17, 2024. The cost data were based on the Japanese public healthcare system.

### Cost-effectiveness analysis including sensitivity analyses

Cost-effectiveness was assessed by comparing the costs of each sedation regimen with its effectiveness in preventing sedation failures, using the ICER.

Deterministic and probabilistic sensitivity analyses were conducted to account for data uncertainty. The primary sources of uncertainty in the model included procedure costs (including sedation procedure fees), sedation success rates, drug costs, MRI duration, and other factors. A Monte Carlo simulation was performed to account for uncertainty in the cost-effectiveness estimates, running 10,000 iterations to generate probability distributions for each anesthesia regimen. Cost-effectiveness acceptability curve (CEAC) and frontier (CEAF) were used to assess the probability of cost-effectiveness at various cost-effectiveness thresholds ranging from $0 to $3500 per aSF.

### Software used

Meta-analyses were conducted using R version 4.4.3; R Foundation for Statistical Computing, Vienna, Austria) with the “meta” package to obtain the success rate of each sedation or GA regimen (Supplementary Materials). Cost-effectiveness analysis was performed using TreeAge Pro Healthcare 2024 (TreeAge Software, LLC, Williamstown, MA, USA).

### Ethics approval

As this study utilized publicly available data and did not involve direct patient interaction or the collection of new clinical data, approval from an institutional ethics review board was waived.

## Results

### Base case analysis

**Fig. 1:**
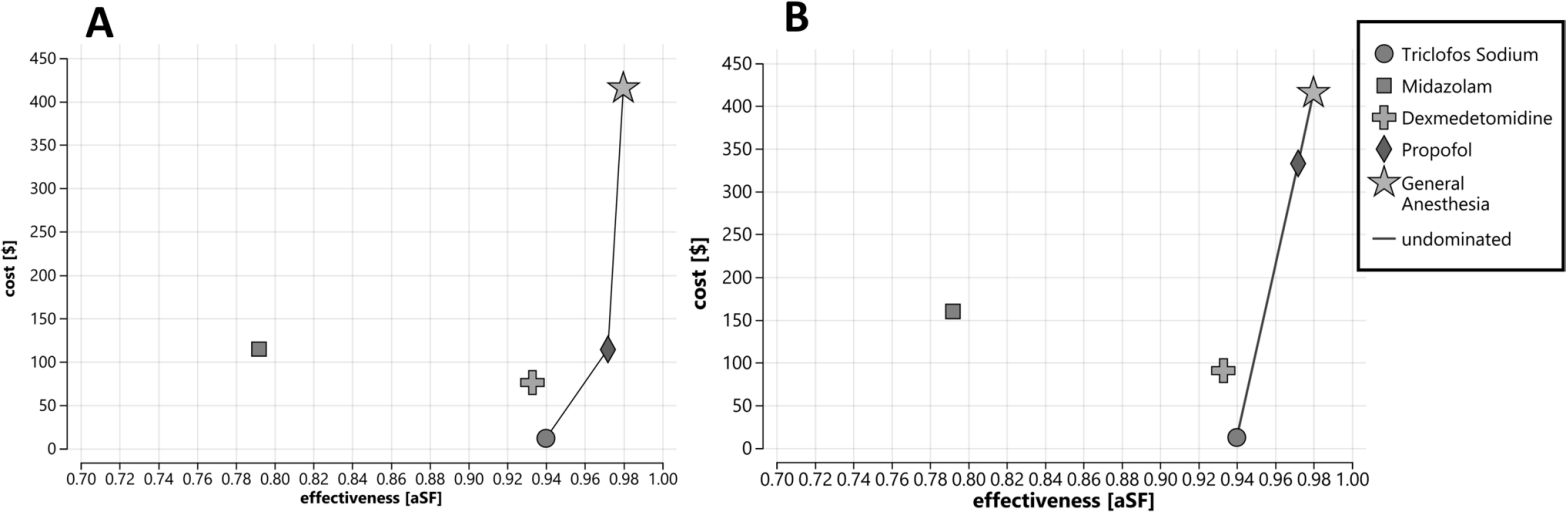
Cost-effectiveness plane for base case analysis comparing all five regimens. **A** The line connecting oral triclofos sodium, IV propofol, and general anesthesia represents the cost-effectiveness boundary, known as the efficiency frontier. The area to the left of the frontier indicates reduced cost-effectiveness. **B** The figure shows the base case analysis comparing all five regimens if the reimbursement for sedation procedures by anesthesiologists were increased from the current $71.50 to $270.00. The line connecting oral triclofos sodium, IV dexmedetomidine, IV propofol, and general anesthesia represents the cost-effectiveness boundary, known as the efficiency frontier. *aSF* averted sedation failure, *IV* intravenous

The cost-effectiveness plane demonstrated the efficiency frontier connecting triclofos sodium, propofol, and GA (Fig. 1A). These regimens displayed a trade-off between cost and effectiveness. Triclofos sodium exhibited the lowest total cost ($11.41) and a high level of effectiveness (0.940). When compared to triclofos sodium sedation by non-anesthesiologists, propofol, administered by anesthesiologists, was more expensive ($114.46) but provided a higher level of effectiveness (0.972), yielding an ICER of $3214.06 per additional unit of effectiveness. Dexmedetomidine sedation administered by non-anesthesiologists was absolutely dominated by chloral hydrate and propofol, with an ICER (−$9222.85) despite being less expensive ($79.95) than propofol and GA. Midazolam, with lower effectiveness (0.792) and higher cost ($114.46), was absolutely dominated by all other regimens, demonstrating a negative incremental effect and an ICER of −$696.28, which indicated it was the least cost-effective sedation option (Table 2).

**Table 2:** Base case analysis

Regimen	Cost [$]	Incr. cost (vs. triclofos sodium) [$]	Effectiveness [aSF]	Incr. effectiveness (vs. triclofos sodium) [aSF]	ICER (vs. triclofos sodium) [$/aSF]	
Triclofos sodium (oral)	11.41	–	0.940	–	–	Undominated
Midazolam (IV)	114.46	103.05	0.792	−0.148	−696.28	Abs. dominated
Dexmedetomidine (IV)	75.97	64.56	0.933	−0.007	−9222.85	Abs. dominated
Propofol (IV)	114.26	102.85	0.972	0.032	3,214.06	Undominated
General anesthesia	415.18	403.77	0.980	0.040	10,094.25	Undominated

*abs.* absolutely, *aSF* averted sedation failure, *Incr.* incremental, *ICER* incremental cost-effectiveness ratio, *IV* intravenous

### One-way sensitivity analysis

The one-way sensitivity analysis identified the procedural fee for sedation by anesthesiologists as the most influential parameter in determining the cost-effectiveness of propofol when compared to dexmedetomidine (Supplementary Fig. 3), triclofos sodium (Supplementary Fig. 4), and midazolam (Supplementary Fig. 5). The sedation success rates of each regimen also significantly impacted the cost-effectiveness results. For the comparison between propofol and dexmedetomidine, the analysis was most sensitive to the reimbursement for sedation procedures by anesthesiologists, following the success rates for each medication.

If the reimbursement for sedation procedures by anesthesiologists were increased from the current $71.50 to $270.00, the ICER for propofol relative to triclofos sodium would become similar to that of GA relative to triclofos sodium (ICER: $10,010.00 vs. $10,072.25, respectively) (Table 3; Fig. 1B).

**Table 3:** Base case analysis when reimbursement rate for sedation procedure by anesthesiologists is $270.00

Regimen	Cost [$]	Incr. cost (vs. triclofos sodium) [$]	Effectiveness [aSF]	Incr. effectiveness (vs. triclofos sodium) [aSF]	ICER (vs. triclofos sodium) [$/aSF]	
Triclofos sodium (oral)	12.29	–	0.940	–	–	Undominated
Midazolam (IV)	159.87	147.58	0.792	−0.148	−997.19	Abs. dominated
Dexmedetomidine (IV)	90.60	78.31	0.933	−0.007	−11,187.14	Abs. dominated
Propofol (IV)	332.61	320.32	0.972	0.032	10,010.00	Undominated
General anesthesia	415.18	402.89	0.980	0.040	10,072.25	Undominated

*abs.* absolutely, *aSF* averted sedation failure, *Incr.* incremental, *ICER* incremental cost-effectiveness ratio, *IV* intravenous

### Probabilistic sensitivity analysis

**Fig. 2:**
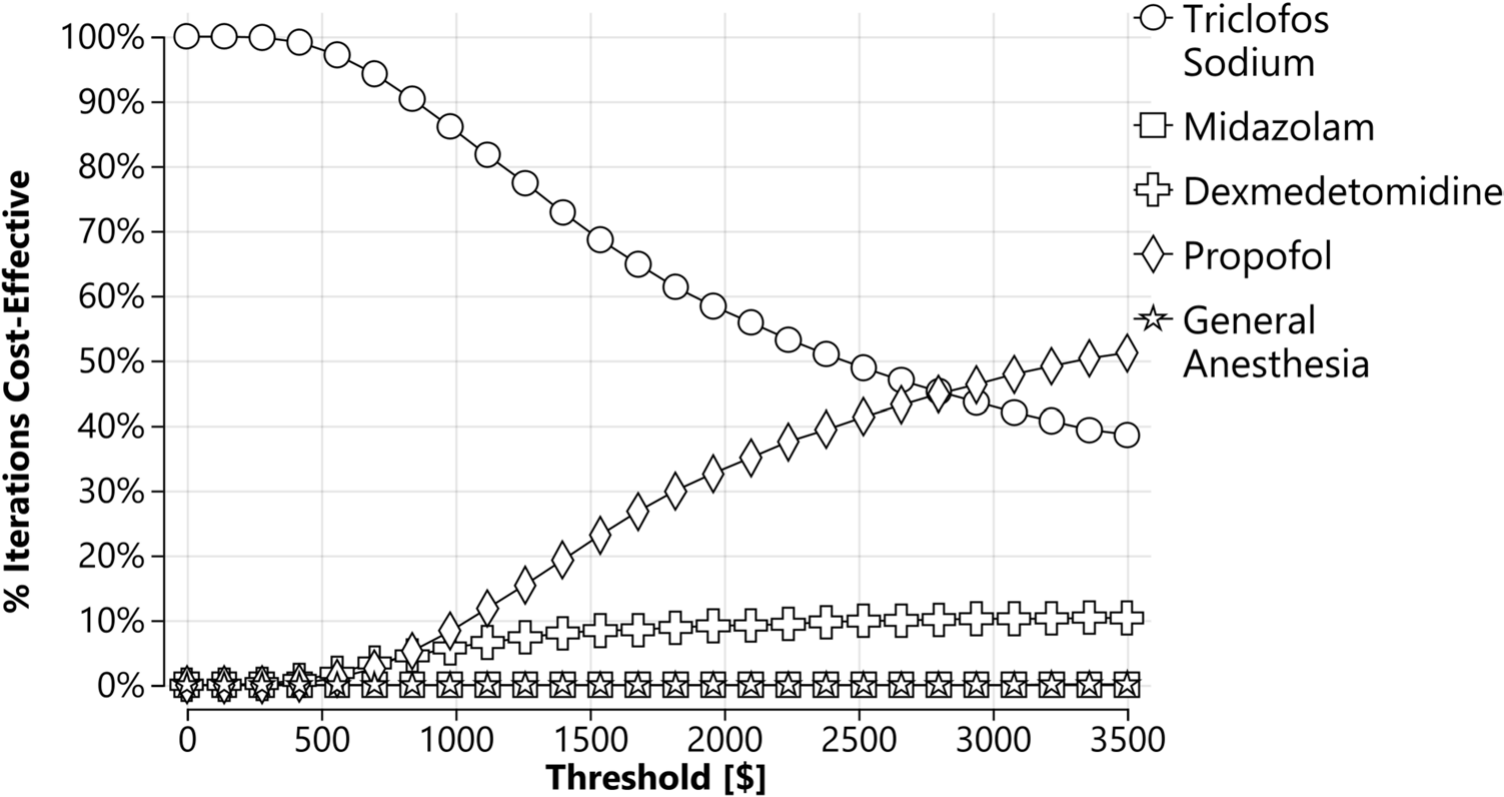
Cost-effectiveness acceptability curves for probabilistic sensitivity analysis comparing all five regimens. The acceptable regimen varies depending on the willingness-to-pay threshold

The probabilistic sensitivity analysis shows the distribution of simulated cost-effectiveness results for each sedation regimen (Supplementary Fig. 6). The analysis confirmed the findings of the base case analysis. Both triclofos sodium and propofol were the most favorable regimens, while midazolam and GA were less favorable from a cost-effectiveness perspective. The CEACs further supported these conclusions, confirming that triclofos sodium and propofol were the most favorable regimens, while midazolam and dexmedetomidine were less favorable (Fig. 2). The probability of propofol being the most cost-effective option varied from 0 to 51.6% at a various threshold ranging from $0 to $3500.00 per aSF, whereas the probability of triclofos sodium being the most cost-effective ranged from 100.0 to 38.9% (Fig. 2).

## Discussion

To our knowledge, this is the first simulation-based cost-effectiveness analysis (CEA) of sedation regimens for pediatric MRI from the Japanese public healthcare system. Our analysis integrates both deterministic and probabilistic sensitivity analyses to ensure robustness. The results suggest that propofol sedation administered by anesthesiologists is more cost-effective than IV midazolam or dexmedetomidine sedation by non-anesthesiologists. This is consistent with prior studies that favor propofol due to its short-acting nature and cost-effectiveness. For example, a U.S. study on pediatric forearm fracture manipulation found that the propofol/fentanyl regimen was the most cost-effective, followed by axillary block and ketamine/midazolam combinations [16].

As healthcare shifts from evidence-based to value-based care, there is an increasing expectation for physicians to deliver high-quality care that incorporates patient values, evidence, and health economics [17]. Our study suggests that improving sedation success rates, either by enabling non-anesthesiologists to safely administer propofol or by increasing anesthesiologist involvement, could enhance the cost-effectiveness of pediatric MRI sedation. This could be achieved through collaboration among anesthesiologists, specialists, and regulatory bodies to establish safe practices and training protocols [2, 18]. Financial incentives can play a crucial role in supporting the time and resources needed for anesthesiologists’ involvement. Increasing reimbursement for sedation procedures by anesthesiologists may incentivize greater participation. Our sensitivity analysis shows that raising reimbursement from $71.50 to $270.00 may be justifiable, given the cost-effectiveness of propofol sedation compared to GA, both of which are assumed to be provided by anesthesiologists. This provides a policy recommendation to increase reimbursement rates for sedation procedures by anesthesiologists.

However, we acknowledge that raising reimbursement rates may not be a sustainable long-term solution, particularly given the anesthesia workforce shortage, especially outside the OR [2, 19]. While such an increase may address short-term challenges, it may not effectively mitigate the long-term demand for sedation and analgesia outside the OR. A promising alternative could be the creation of a dedicated sedation team, involving anesthesiologists, pediatric specialists, and nurses. Previous studies support the idea that dedicated sedation teams improve safety and procedural success rates. For instance, a pediatric sedation service resulted in fewer incomplete studies (2.7 to 0.8%) and fewer cancelations due to patient illness (3.8 to 0.6%), as well as reduced hypoxia rates (8.8 to 4.6%) and fewer referrals for GA (2.1 to 0.1%) [20]. Another study reported that a dedicated sedation team performed 784 pediatric procedural sedations in one year, primarily using propofol (79%) and esketamine for painful procedures (48%). Despite a high-risk population (12.2% infants under 1 year, 41.9% ASA-PS III or IV), the team achieved a low adverse event rate (6.5%), with low professional experience and increased propofol dosage as risk factors. This highlights the safety and the efficacy of a dedicated sedation team in high-risk pediatric procedures outside the OR [21]. These findings highlight that a team-based approach could help avoid the cost burden while maintaining safety and efficacy.

Based on these findings, one potential solution to address the anesthesia workforce shortage would be to redirect increased reimbursement rates for sedation by anesthesiologists (from $71.50 to $270.00, which our results suggest is justifiable) to support the formation and operation of dedicated sedation teams. By supporting a team-based approach, healthcare systems could deliver high-quality, cost-effective sedation services, while also mitigating the burden of the anesthesia workforce shortage. Such an approach could help balance demand and supply as anesthesiologists are needed both in and outside the OR. It is important to note, however, that financial incentives alone may not drive the desired improvements. Behavioral economics suggests that financial incentives must align with broader goals of quality care and teamwork to be effective [22]. Therefore, further research using real-world data and standardized methodologies will be necessary to fully evaluate the implications of such policy changes and refine strategies for optimal team-based care.

We assumed that propofol sedation would be administered by anesthesiologists, consistent with typical pediatric sedation practices in Japan [5]. The use of propofol by non-anesthesiologists for procedural sedation remains controversial [13]. Some studies suggest that non-anesthesiologists can safely administer propofol with proper training and protocols [23], while others argue that it should be restricted to anesthesia professionals due to potential side effects [13]. This debate is especially relevant in pediatric sedation, where concerns persist about the increasing use of propofol by non-anesthesiologists, despite evidence supporting its safety in appropriately selected cases [23]. European guidelines for non-anesthesiologist propofol administration in gastrointestinal endoscopy have faced opposition from anesthesiology societies [13]. This controversy highlights the growing demand for sedation outside the OR and the need for effective, high-quality care.

Recent studies on the combination of propofol and dexmedetomidine for pediatric procedural sedation have shown promising results, including fewer airway complications and lower rates of desaturation compared to propofol alone [24]. A trial evaluating dexmedetomidine for pediatric MRI sedation found that propofol rescue was needed in 36.8% of cases in the high-dose dexmedetomidine group, compared to 85.7% in the low-dose group [12]. This suggests that propofol can serve as an effective rescue agent in dexmedetomidine sedation, further justifying its combination with other sedatives for pediatric MRI procedures. Studies also show that with proper training and monitoring, non-anesthesiologists can safely administer propofol for certain procedures [25]. Although sedation success ultimately depends on the provider’s skills and the clinical context, not just pharmacological properties [18], our study suggests that the use of propofol may be justified from a cost-effectiveness perspective, especially if it helps non-anesthesiologists achieve higher sedation success rates.

Our prior CEA, based on clinical data from a single institution, indicated that propofol sedation by anesthesiologists was more cost-effective than oral or rectal sedation (including triclofos sodium and chloral hydrate) by non-anesthesiologists for pediatric MRI in Japan. In contrast, the current analysis suggests that triclofos sodium sedation by non-anesthesiologists would be the most cost-effective. This change is due to updated success rate of sedation by triclofos sodium, derived from a meta-analysis [26], which likely provides a more generalizable estimate of its effectiveness. However, triclofos sodium and chloral hydrate have been less favored in other countries due to long half-lives and serious adverse events, including fatalities and neurological complications, especially in neonates [27]. Our analysis did not account for these potential complications or long-term outcomes, which could impact both patient health and healthcare costs. Future analyses should incorporate these factors to fully capture the societal and economic burden of sedation failures.

Our study has several strengths. We used data from a broad range of published literature, including meta-analysis, which enhances the generalizability of our findings. The use of deterministic and sensitivity analyses strengthens the reliability of our results under varying assumptions. While the results may not be directly applicable to healthcare systems outside Japan, the analytical methods employed in this study can serve as a useful model for anesthesiologists and policymakers in other settings.

However, there are several limitations. We modeled a 3-year-old patient with ASA-PS I/II and did not account for patient-specific factors, such as age, ASA-PS class, psychosomatic behavior, or a history of sedation failure, all of which can affect sedation outcomes. Future studies should consider these variables to provide a more accurate reflection of real-world data. Second, simulation-based models have inherent limitations due to assumptions made, and results should be interpreted cautiously. Open-source publication of such models and sensitivity analyses can improve transparency [28]. Third, although we conducted a structured literature search using PubMed to obtain the sedation success rates of each regimen, the search was limited to the single database and conducted by the two co-authors. As a result, some relevant studies may have been missed. Moreover, source studies vary in study designs, patient populations, and definitions of success. While we selected studies with comparable age groups and procedural contexts, heterogeneity remains, which may have influenced the pooled estimates. To address this uncertainty, we conducted both deterministic and probabilistic sensitivity analyses to test the robustness of our findings under varying assumptions. Finally, while we used aSF as the primary effectiveness measure, it may not fully capture the broader impact of sedation failures on patients' quality of life (QOL). Future studies should include QOL measures, such as quality-adjusted life years (QALYs) to provide a more comprehensive evaluation of sedation failure impacts.

## Conclusion

This simulation-based cost-effectiveness analysis suggests that propofol sedation administered by anesthesiologists would be more cost-effective than dexmedetomidine or midazolam sedation provided by non-anesthesiologists, primarily due to relatively low reimbursement rate for sedation procedures by anesthesiologists. These findings indicate that increasing reimbursement for sedation procedures by anesthesiologists may be justified for increase in direct involvement of anesthesiologists in sedation for pediatric MRI, which may lead to better cost-effectiveness. However, further real-world validation is necessary to assess the broader applicability and long-term implications of these findings for healthcare policymaking.

## Supplementary Information

Below is the link to the electronic supplementary material.

Supplementary file1 (DOCX 4392 KB)

## Data Availability

The datasets generated and analyzed during the current simulation study are not publicly available but are available from the corresponding author upon reasonable request.
