# Supplementary material for: Cost-effectiveness analysis of sedation regimens for children undergoing magnetic resonance imaging in Japan: a simulation-based study
Source: J Anesth. 2025 Jul 11;40(2):182–91. doi: 10.1007/s00540-025-03540-8 (PMC13035560; doi:10.1007/s00540-025-03540-8)
Supplement: Supplementary file 1 — Supplementary file1 (DOCX 4392 KB) [file 540_2025_3540_MOESM1_ESM.docx]

**Title: Cost-effectiveness analysis of sedation regimens for children undergoing magnetic resonance imaging in Japan: a simulation-based study**

**Authors:** Soichiro Obara, M.D., Dr.P.H., Yoshinori Nakata, M.D., Ph.D.

**Affiliation:**

Teikyo University Graduate School of Public Health, Tokyo, Japan

Postal Address: 2-11-1 Kaga, Itabashi-ku, Tokyo, 173-8605, JAPAN

Phone: +81-3-3964-1211 (extension 46225)

(Soichiro Obara & Yoshinori Nakata)

**Corresponding to:** Soichiro Obara, M.D., Dr.P.H.

**E-mail address:** obara.souichirou.bv@teikyo-u.ac.jp; soichoba1975@gmail.com

**Supplementary Materials**

**Table of Contents Page**

**Supplementary Figure 1 (Decision tree model) ....................................................................................................................................................... 3**

**Supplementary Figure 2 (Decision tree model used in the study) ……………………………………………………………………………………... 4**

**Supplementary Figure 3 (Tornado diagram for one-way sensitivity analysis comparing the ICER of dexmedetomidine vs. propofol) ……... 8**

**Supplementary Figure 4 (Tornado diagram for one-way sensitivity analysis comparing the ICER of triclofos sodium vs. propofol) ………….. 9**

**Supplementary Figure 5 (Tornado diagram for one-way sensitivity analysis comparing the ICER of midazolam vs. propofol) ..................... 10**

**Supplementary Figure 6 (Cost-effectiveness plane for probabilistic sensitivity analysis comparing all five regimens) …............................ 11**

**Supplemental method: Meta-analysis of the success rate for each sedation or general anesthesia regimen ………………………………...…… 12**

**Supplementary References (References 52–67) ……………………………………………………………………………………………………………... 19**

**Supplementary Figure 7 (PRISMA flow diagram) and its footnote ……………………………………………………………………………………... 21**

**Supplementary Figure 8-A (Forest plot comparing eligible studies on intravenous midazolam) ……………......................................................... 22**

**Supplementary Figure 8-B (Forest plot comparing eligible studies on intravenous propofol) ……………......................................................... 23**

**Supplementary Figure 8-C (Forest plot comparing eligible studies on general anesthesia) ……………......................................................... 24**

**CHEERS 2022 Checklist ……………………………………………………………………………………………………………………………………………... 25**

**Supplementary Figure 1: Decision tree model**


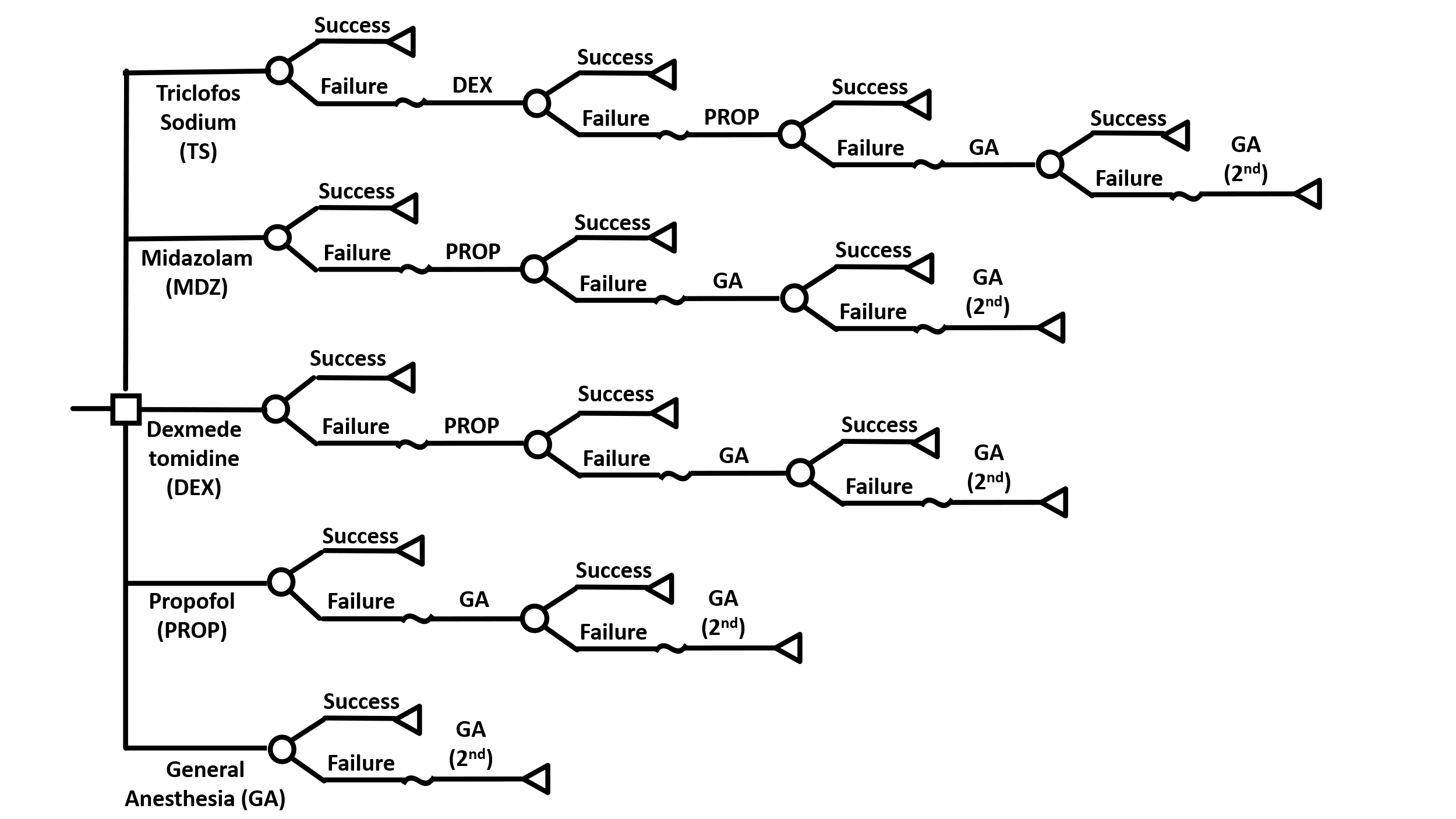


**Legend: Graphical notation in the DT model:**

□ **Decision node**: Indicates a clinical decision point (i.e., choice of initial sedation regimen).

**○ Chance node**: Represents a probabilistic event (i.e., sedation success or failure).

△ **Terminal node**: Represents a final outcome, associated with its cost and effectiveness (i.e., averted sedation failure).

**Supplementary Figure 2. Decision tree model used in the study**


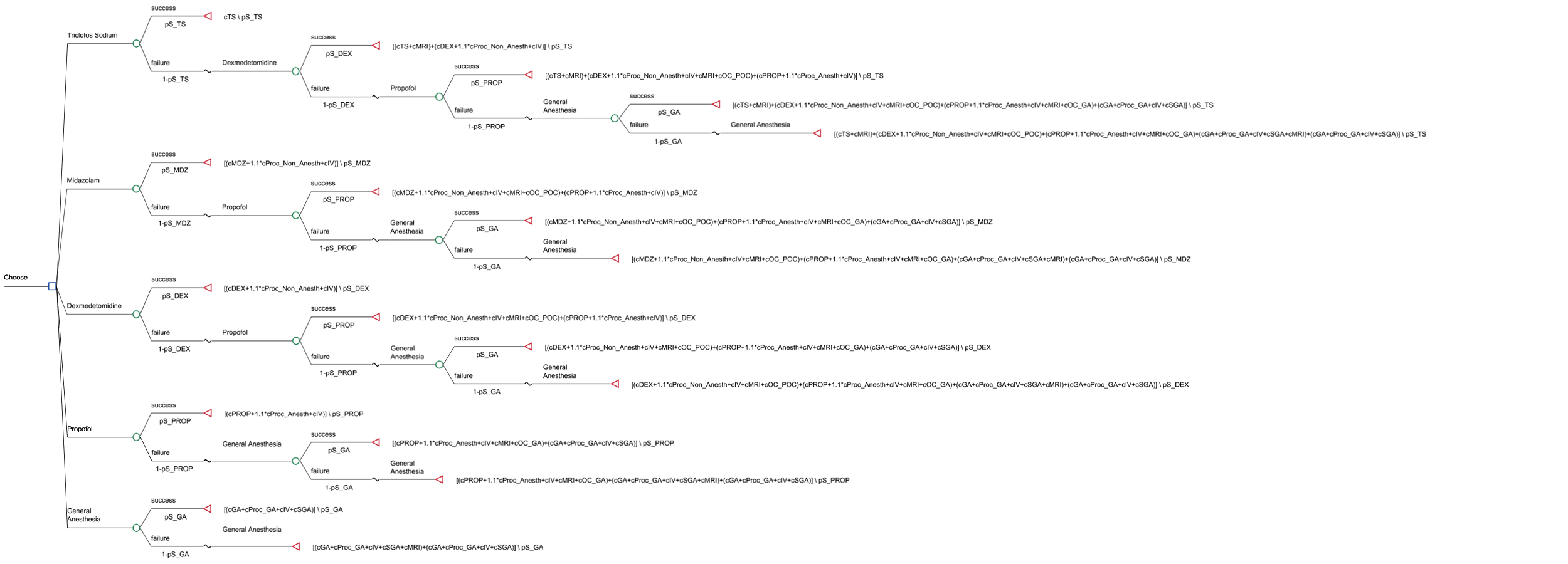


**Legend:**

This diagram presents the decision tree (DT) model used to evaluate the cost-effectiveness of various sedation and anesthesia regimens for pediatric MRI in Japan. The model was developed using TreeAge Pro Healthcare 2024 software (TreeAge Pro Inc., Williamstown, MA, USA).

**Graphical notation in the DT model:**

□ **Decision node**: Indicates a clinical decision point (i.e., choice of initial sedation regimen).

○ **Chance node**: Represents a probabilistic event (i.e., sedation success or failure).

△ **Terminal node**: Represents a final outcome, associated with its cost and effectiveness (i.e., averted sedation failure).

All abbreviated variables (e.g., pS_PROP, cPROP) are defined in the below.

**Decision tree interpretation:**

1. Each branch resprensts a potential path.
2. At each chance noted, probabilities (e.g. pS_PROP for successful IV propofol sedation) are assigned.
3. Each terminal node reflects a cost and outcome pair. For example, the total cost for failed IV propofol sedaiton folloed by successful general anesthesia includes medication (cPROP), procedural fee (cPROP_Anesth), IV access (cIV), MRI scan fee (cMRI), related opportunity costs (cOC_GA) and procedural fee for general anesthesia fee (cGA) are included.
4. Expected values are automatically calculated by the software based on probabilities and payoffs provided.

**Model structure and assumptions:** The decision tree captures the sequential pathway of sedation regimens following initial failure, based on routine clinical practices in Japan and our previous institutional study:

1. If oral triclofos sodium fails, IV dexmedetomidine is administered by non-anesthesiologists.
2. If IV dexmedetomidine or midazolam fails, IV propofol is administered by anesthesiologists.
3. If IV propofol fails, general anesthesia with inhalational sevoflurane is used with a supraglottic airway (e.g., i-gel).
4. If general anesthesia fails, a second attempt is assumed to succeed.

The model does not use a Markov structure, as it addresses a single, non-recurring decision point (i.e., whether sedation succeeds or fails). The primary outcome is the prevention of sedation failure, expressed as “averted sedation failure (aSF)”, calculated as: aSF = 1 − sedation failure rate = 1 − (1 − sedation success rate).

**Costing perspective and elements:** The analysis was conducted from the perspective of the Japanese public healthcare payer, including direct healthcare costs but excluding indirect costs, such as family productivity losses due to sedation failure. Direct costs considered in the analysis were the costs of medications, costs of devices, sedation and general anesthesia procedure fees, and opportunity costs incurred when sedation fails. Since the Japanese national medical fee schedule reimburses hospital fees but not individual doctor’s fees, staff time costs were estimated based on procedure costs, using medical remuneration points.

**Additional assumptions:** In the case of MRI being aborted due to sedation failure, additional costs were considered as opportunity costs: (1) MRI scan: The cost of MRI scan was considered as an opportunity cost, assuming that the aborted scan could be reallocated to another patient for the time frame. (2) Non-anesthesiologist-administered sedation failure: If IV sedation by a non-anesthesiologist (midazolam or dexmedetomidine) failed, pediatric outpatient consultation fees for non-anesthesiologists were added as an opportunity cost. (3) Anesthesiologist-administered sedation failure: If IV sedation by an anesthesiologist (propofol) failed, general anesthesia fees were added as an opportunity cost, assuming that an anesthesiologist would have provided general anesthesia in the OR, had they not been involved in the sedation outside the OR.

When IV sedation is administered to children aged 3 to under 6 years, an additional fee, known as the "infant add-on," is applied. This add-on is calculated by adding 10% of the prescribed point value to the standard point value for the sedation procedure. Specifically, the reimbursement rate is increased by an amount equivalent to 10% of the standard points for each relevant procedure. All patients are assumed to be discharged the same day without complications, regardless of success or failure.

**Abbreviations:**

cDEX: Cost of sedation with intravenous dexmedetomidine

cGA: Cost of general anesthesia with sevoflurane

cIV: Procedure fee for securing intravenous line plus cost of intravenous fluids

cMCP: Cost of intravenous metoclopramide

cMDZ: Cost of sedation with intravenous midazolam

cMRI: MRI fee (The cost of MRI scan was considered as an opportunity cost, assuming that the aborted scan the MRI could be reallocated to another patient for the time frame.)

cOC_GA: Opportunity cost for failed anesthesia-led propofol sedation (If intravenous sedation by an anesthesiologist (propofol) failed, general anesthesia fees were added as an opportunity cost)

cOC_POC: Opportunity cost for outpatient consultation (Fee for pediatric outpatient clinic)

cProc_Anesth: Procedure fee for sedation administered by anesthesiologists

cProc_GA: Procedure fee for general anesthesia

cProc_Non_Anesth: Procedure fee for sedation administered by non-anesthesiologists

cPROP: Cost of sedation with intravenous propofol

cSGA: Cost of supraglottic airway device

cTS: Cost of sedation with oral triclofos sodium

pS_TS: Probability of success of sedation with oral triclofos sodium

pS_DEX: Probability of success with intravenous dexmedetomidine

pS_MDZ: Probability of success with intravenous midazolam

pS_PROP: Probability of success with intravenous propofol

pS_GA: Probability of success with general anesthesia

**Supplementary Figure 3. Tornado diagram for one-way sensitivity analysis comparing the ICER of dexmedetomidine vs. propofol**


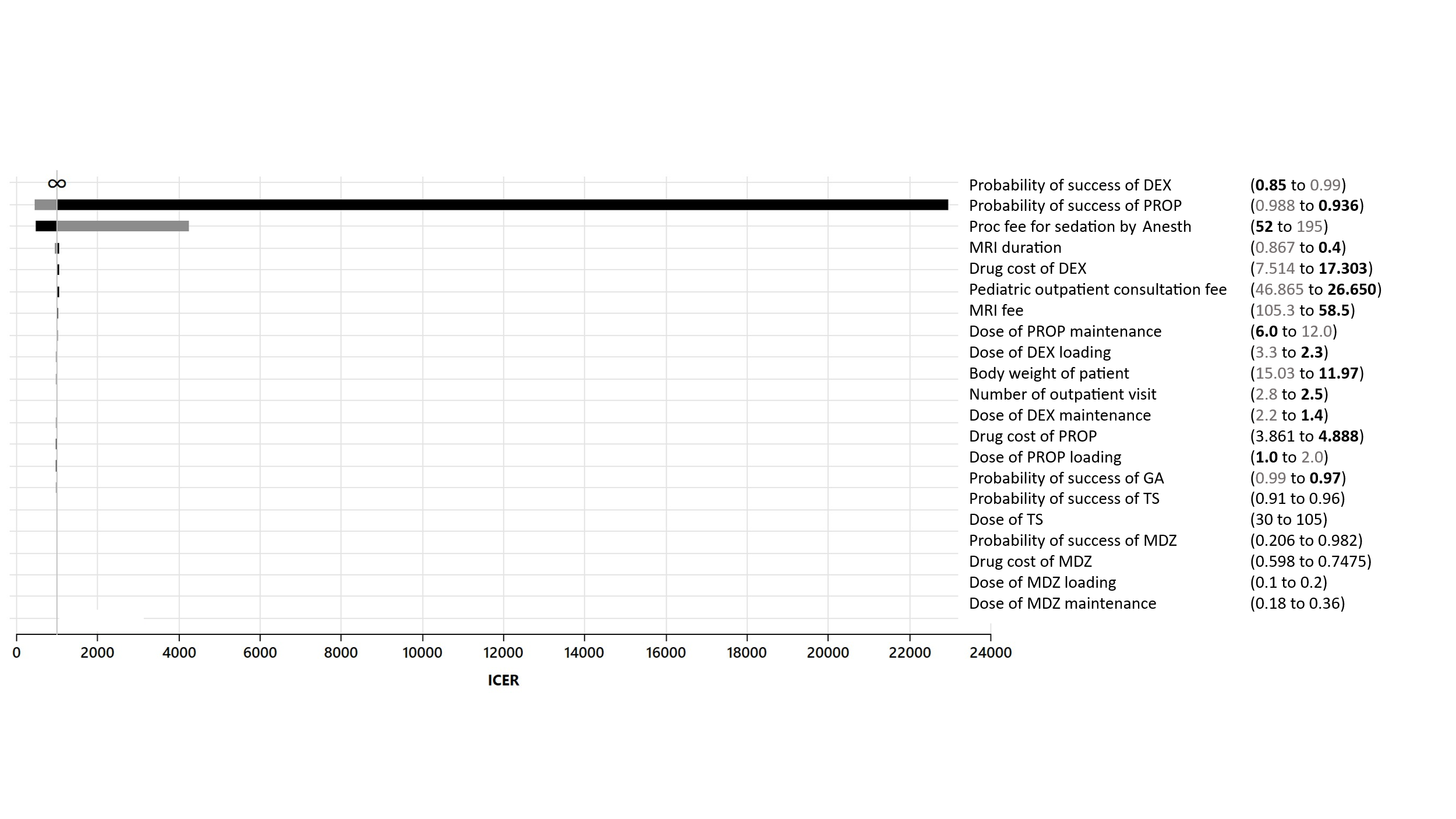


**Legend:** The procedural fee for sedation by anesthesiologists was the most influential parameter affecting cost-effectiveness.

**Abbreviations:** Anesth: Anesthesiologists, DEX: dexmedetomidine, EV: expected value, ICER: incremental cost-effectiveness ratio, MDZ: midazolam, MRI: magnetic resonance imaging, Proc: procedure, PROP: propofol, TS: tricofos sodium

**Supplementary Figure 4. Tornado diagram for one-way sensitivity analysis comparing the ICER of triclofos sodium vs. propofol**


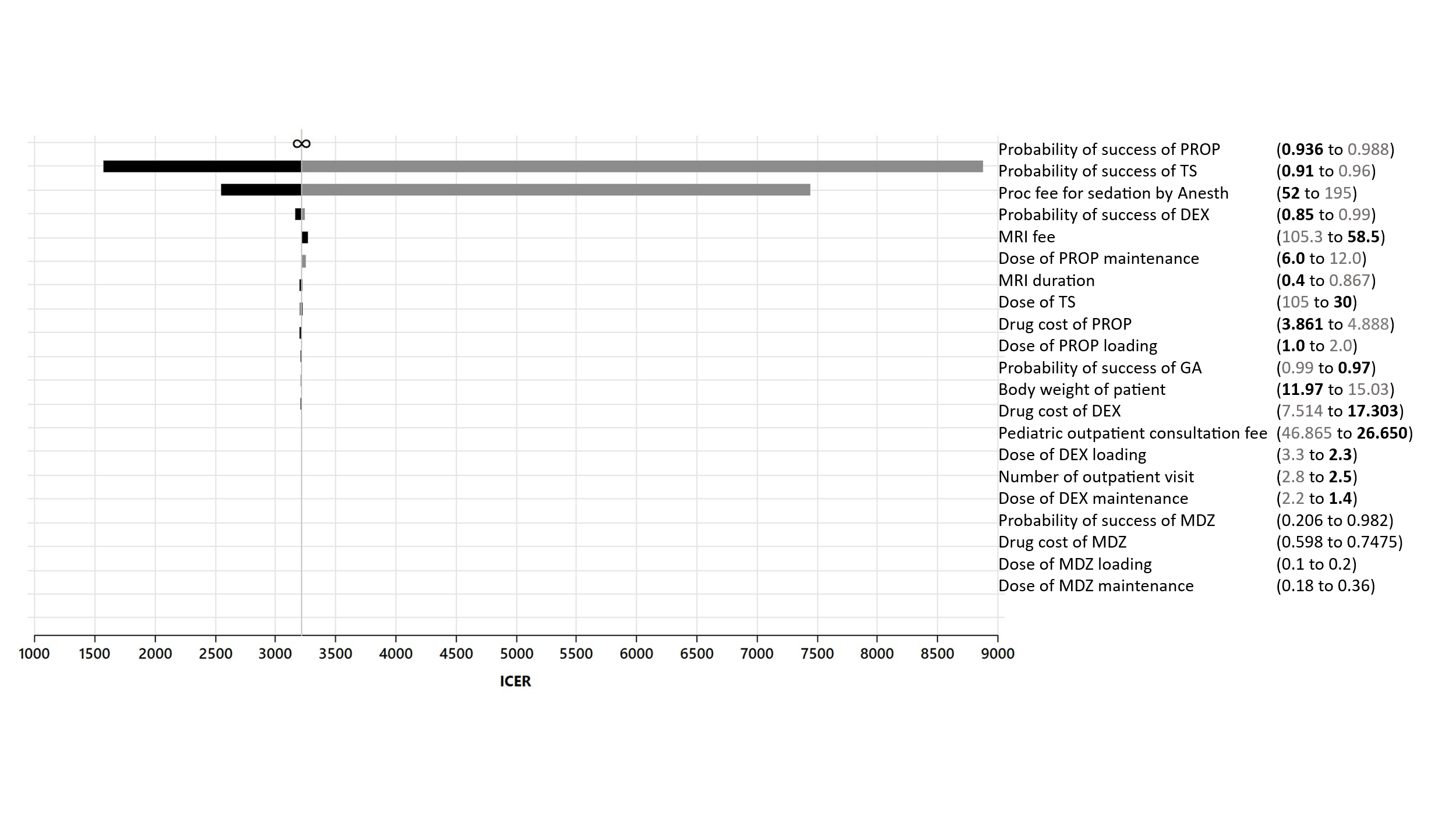


**Legend:** The procedural fee for sedation by anesthesiologists was the most influential parameter affecting cost-effectiveness.

**Abbreviations:** Anesth: anesthesiologists, DEX: dexmedetomidine, EV: expected value, ICER: incremental cost-effectiveness ratio, MDZ: midazolam, MRI: magnetic resonance imaging, Proc: procedure, PROP: propofol, TS: triclofos sodium

**Supplementary Figure 5. Tornado diagram for one-way sensitivity analysis comparing the ICER of midazolam vs. propofol**


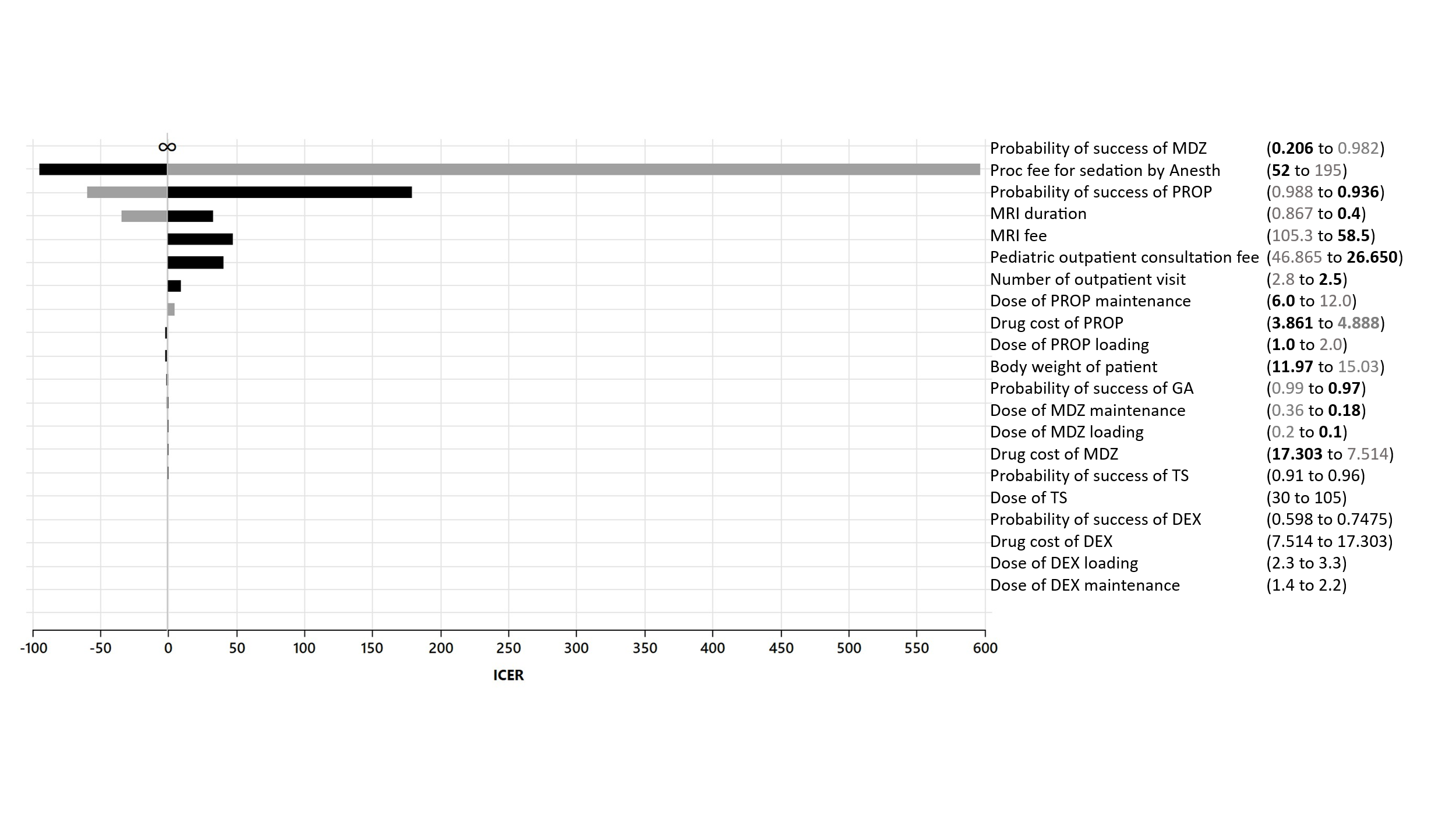


**Legend:** The procedural fee for sedation by anesthesiologists was the most influential parameter affecting cost-effectiveness.

**Abbreviations:** Anesth: anesthesiologists, DEX: dexmedetomidine, EV: expected value, ICER: incremental cost-effectiveness ratio, MDZ: midazolam, MRI: magnetic resonance imaging, Proc: procedure, PROP: propofol, TS: triclofos sodium

**Supplementary Figure 6. Cost-effectiveness plane for probabilistic sensitivity analysis comparing all five regimens**


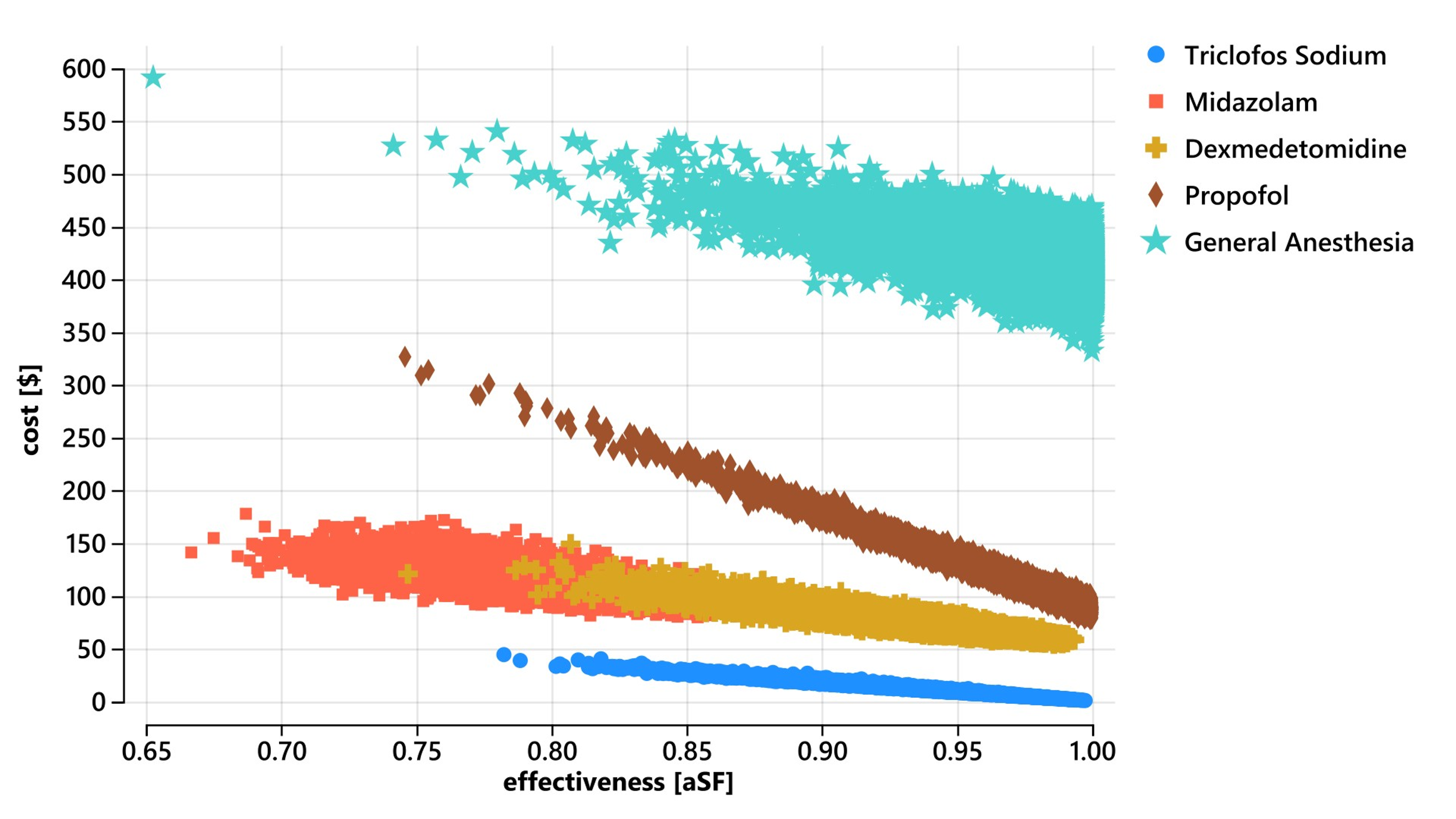


**Supplemental method: Meta-analysis of the success rate for each sedation or general anesthesia regimen**

**Objective:**

To estimate averted sedation failure of sedation regimens, success probabilities were derived from multiple published studies.

**Method:**

We conducted a literature search to inform the input parameters of success rate of sedation or general anesthesia for this simulation-based cost-effectiveness analysis. The search was performed in PubMed using the following search string:

**("Pediatric" OR "Children" OR "Child") AND ("Magnetic Resonance Imaging" OR "MRI") AND ("Sedation" OR "Anesthesia") AND ("Efficacy" OR "Success" OR "Failure" OR "Failed") AND ("Chloral hydrate" OR "Triclofos" OR "Midazolam" OR "Dexmedetomidine" OR "Propofol" OR "General Anesthesia")**

Filters were applied to restrict the results to articles published in English between 2000 and 2024.

Two authors (S.O. and Y.N.) jointly screened the titles and abstracts, followed by full-text assessments to identify studies that met the predefined eligibility criteria. The selection process was performed collaboratively, rather than independently, due to the pragmatic aim of identifying suitable input parameters rather than conducting a formal meta-analysis.

Inclusion criteria were as follows: (1) participants aged ≦20 years; (2) sedation or anesthesia with one of the following monotherapies: oral chloral hydrate, oral triclofos, intravenous midazolam, intravenous dexmedetomidine, intravenous propofol, or general anesthesia; (3) availability of quantitative data on the success rate of the sedation or anesthesia regimen; (4) study design categorized as a clinical trial, observational study, randomized controlled trial, systematic review, or meta-analysis.

Exclusion criteria included: (1) studies with participants aged >20 years; (2) use of combined or multimodal sedative regimens (without monotherapy data); (3) basic or preclinical animal research; (4) publications in the form of abstracts, letters, case reports, editorials, notes, methods papers, or protocols; (5) articles not published in English.

Systematic reviews and meta-analyses were included in the search strategy because they can provide synthesized and potentially more robust estimates of sedation success rates, which are essential as model inputs for cost-effectiveness analysis. When multiple meta-analyses were identified, the most appropriate source was selected based on recency, methodological quality, and relevance to the study population and interventions of interest.

**Results:**

A total of 212 records were identified through PubMed. After title and abstract screening, 132 were excluded for low relevance. The full texts of the remaining 80 articles were assessed for eligibility. Of these, 40 were excluded due to unclear data or inconclusive results, and 7 were excluded for not reporting monotherapy sedation outcomes and for duplicated reports. During the full-text screening process, two additional eligible studies were identified through reference screening of included meta-analyses. Although this study was not retrieved through the original PubMed search strategy, it met all predefined inclusion criteria and provided important comparative data on midazolam and propofol monotherapy. Therefore, those were added during the eligibility assessment phase, bringing the total number of included studies to 35. Finally, 35 studies were included in the review and used to inform model input parameters.

The study selection process is summarized in a PRISMA flow diagram (**Supplementary Figure 7**).

These studies reported sedation success rates for at least one of the following monotherapy regimens used in pediatric MRI: oral triclofos sodium or chloral hydrate, intravenous midazolam, intravenous dexmedetomidine, intravenous propofol, or general anesthesia. It should be noted that some studies reported outcomes for more than one sedation regimen, resulting in overlapping inclusion across categories.

The distribution of included studies by sedation regimen and study design was as follows:

1. **Oral triclofos sodium or chloral hydrate**: 1 meta-analysis [26], 1 randomized trial [67], and 8 observational studies [5,52,53,57,58,61,64,66]
2. **Intravenous midazolam**: 3 observational studies [5,32,36]
3. **Intravenous dexmedetomidine**: 3 meta-analyses [33,54,55], 1 randomized trial [12], and 11 observational studies [32,37,39,40,47,56,59,60,62,63,65]
4. **Intravenous propofol**: 2 meta-analyses [10,12], 1 randomized trial [28], and 11 observational studies [5,37-46]
5. **General anesthesia**: 1 randomized trial [48] and 4 observational studies [5,36,47,49]

Meta-analyses were included when available, as they provide synthesized estimates with greater statistical power, enhanced generalizability, and reduced uncertainty.

These studies were used to extract sedation success rates as follows:

1. **Oral triclofos sodium or chloral hydrate**

For oral chloral hydrate or triclofos sodium, our final review identified one recent meta-analysis as well as nine observational studies. To ensure the use of the most robust and generalizable estimate for our simulation-based cost-effectiveness model, we adopted the pooled sedation success rate reported in the meta-analysis by de Rover et al. (2023) [26], which systematically reviewed needle-free pharmacological sedation techniques in pediatric patients undergoing imaging procedures. This study synthesized data from multiple eligible studies on chloral hydrate and reported a pooled success rate of **94.0% (95% CI: 91.0%–96.0%; I² = 98%; τ^2^=1.5228; p < 0.01)**. Although considerable heterogeneity was observed, this meta-analysis provided a comprehensive and statistically rigorous estimate based on a large aggregated sample. Therefore, this pooled estimate was deemed more reliable than those from individual observational studies and was used as the input parameter for oral chloral hydrate or triclofos sodium in the simulation.

1. **Intravenous midazolam**

The sedation success rates from the included studies were as follows:

- Malviya et al. (2000): 82 successes out of 90 = 82/90 [36]
- Koroglu et al. (2005): 8/40 [32]
- Obara et al. (2022): 293/307 [5]

To estimate the sedation success rate of intravenous midazolam, we conducted a meta-analysis of 3 observational studies using a random-effects model with logit transformation and inverse-variance weighting. A continuity correction of 0.5 was applied to studies reporting a 100% success rate. Between-study heterogeneity was assessed using the I² and τ² statistics. Meta-analysis was performed in R (version 4.4.3) using the meta package.

Across the 3 studies, a total of 437 pediatric patients were included, with 383 successful sedations. The pooled success rate under the random-effects model was **79.2% (95% CI: 20.6%–98.2%)**, with a prediction interval of 0%–100%, indicating very high heterogeneity across studies **(I² = 97.7%, τ² = 5.5064, p < 0.0001)**. For comparison, the common-effect model estimated a pooled success rate of 85.83% (95% CI: 80.60%–89.83%). Given the extremely high heterogeneity, the random-effects estimate was considered more appropriate and was used as the input parameter for intravenous midazolam efficacy in our simulation-based cost-effectiveness analysis.

A forest plot illustrating the individual study estimates along with the pooled success rates is provided as **Supplementary Figure 8-A**.

1. **Intravenous dexmedetomidine**

For intravenous dexmedetomidine, three meta-analyses were identified in our final review. Among these, to ensure the use of the most robust and generalizable estimate for our simulation-based cost-effectiveness model, we adopted the pooled sedation success rate from the more recent and comprehensive meta-analysis by Angelopoulou et al. (2023) [33], which included 838 pediatric patients undergoing MRI. This study provided a clearly defined and clinically relevant endpoint—sedation failure defined as the need for an additional sedative agent—and reported a failure rate of 6.7% (56 out of 838 patients; 95% CI: 1%–15%, I² = 93%, τ² = 0.0454, p < 0.01), corresponding to a success rate of **93.3% (95% CI: 85.0%–99.0%)**. Given its larger sample size, synthesis of multiple trials, clearly defined endpoint, and improved statistical precision, this estimate was considered superior to those derived from individual observational studies or older meta-analyses, and was therefore selected as the input parameter for dexmedetomidine efficacy in the simulation.

1. **Intravenous Propofol**

Although two previous meta-analyses have evaluated the efficacy of intravenous propofol for pediatric procedural sedation (Zou et al., (2017) [54]; Fang et al., (2015) [55]), both were published more than five years ago and included only a limited number of studies reporting sedation success rates, which may limit the statistical stability and current relevance of their findings. Therefore, we conducted an updated meta-analysis using data from 1 randomized trial and 10 observational studies that explicitly reported sedation success as an outcome.

The sedation success rates from the included studies were as follows:

- Amundsen et al. (2005): 106 successes out of 108 = 106/108 [46]
- Koroglu et al. (2006): 27/30 [37]
- Dalal PG et al. (2006): 68/68 [45]
- Persad et al. (2007): 30/30 [44]
- Machata et al. (2008): 500/500 [43]
- Griffiths et al. (2013): 106/109 [42]
- Wu et al. (2014): 48/49 [41]
- Kamal et al. (2017): 25/30 [40]
- Kamat et al. (2018): 49/49 [39]
- Abulebda et al. (2018): 49/49 [38]
- Obara et al. (2022): 26/26 [5]

To estimate the sedation success rate, we conducted a meta-analysis using a random-effects model with logit transformation and inverse-variance weighting. A continuity correction of 0.5 was applied to studies reporting a 100% success rate. Between-study heterogeneity was assessed using the I² and τ² statistics. Meta-analysis was performed in R (version 4.4.3) using the meta package.

Across the 11 studies, a total of 1,048 pediatric patients were included, with 1,034 successful sedations. The pooled success rate under the random-effects model was **97.2% (95% CI: 93.6%–98.8%)**, with a prediction interval of 71.4%–99.8%, indicating moderate-to-substantial heterogeneity across studies **(I²=64.0%, τ²=1.2080, p=0.0019)**. For comparison, the common-effect model estimated a pooled success rate of 95.2% (95% CI: 92.6%–97.0%). Given the heterogeneity, the random-effects estimate was considered more appropriate and was used as the input parameter for intravenous propofol efficacy in our simulation-based cost-effectiveness analysis.

A forest plot illustrating the individual study estimates along with the pooled success rates is provided as **Supplementary Figure 8-B**.

1. **General Anesthesia**

The sedation success rates from the included studies were as follows:

- Malviya et al. (2000): 140 successes out of 140 = 140/140 [36]
- Fogel et al. (2008): 161/161 [49]
- Ogurlu et al. (2010): 93/96 [48]
- Lepeltier et al. (2022): 52/52 [47]
- Obara et al. (2022): 14/14 [5]

To estimate the sedation success rate of general anesthesia, we conducted a meta-analysis of 1 randomized trial and 4 observational studies using a random-effects model with logit transformation and inverse-variance weighting. A continuity correction of 0.5 was applied to studies reporting a 100% success rate. Between-study heterogeneity was assessed using the I² and τ² statistics. Meta-analysis was performed in R (version 4.4.3) using the meta package.

Across the 5 studies, a total of 463 pediatric patients were included, with 460 successful sedations. The pooled success rate under the random-effects model was **98.5% (95% CI: 95.3%–99.5%)**, with a prediction interval of 82.6%–99.9%, indicating low-to-moderate heterogeneity across studies **(I² = 19.2%, τ² = 0.5310, p = 0.2922)**. For comparison, the common-effect model estimated a pooled success rate of 98.0.% (95% CI: 95.4%–99.1%). Given the low heterogeneity, the random-effects estimate was deemed more appropriate and was used as the input parameter for general anesthesia efficacy in our simulation-based cost-effectiveness analysis.

A forest plot illustrating the individual study estimates along with the pooled success rates is provided as **Supplementary Figure 8-C**.

**Supplementary References**

1. Cui Y, Guo L, Mu Q, Kang L, Chen Q, Wu Q, He Y, Tang M. Analysis of Risk Factors for Chloral Hydrate Sedative Failure with Initial Dose in Pediatric Patients: a Retrospective Analysis. Paediatr Drugs. 2022;24:403-12.
2. Moon JU, Han JY. Effectiveness of Chloral Hydrate on Brain MRI in Children with Developmental Delay/Intellectual Disability Comparing with Normal Intelligence: Single Tertiary Center Experience. Children (Basel). 2021;8:1097.
3. Zhou Q, Shen L, Zhang X, Li J, Tang Y. Dexmedetomidine versus propofol on the sedation of pediatric patients during magnetic resonance imaging (MRI) scanning: a meta-analysis of current studies. Oncotarget. 2017;8:102468-73.
4. Fang H, Yang L, Wang X, Zhu H. Clinical efficacy of dexmedetomidine versus propofol in children undergoing magnetic resonance imaging: a meta-analysis. Int J Clin Exp Med. 2015;8:11881-9.
5. Ahmed SS, Unland T, Slaven JE, Nitu ME. High dose dexmedetomidine: effective as a sole agent sedation for children undergoing MRI. Int J Pediatr. 2015;2015:397372. Erratum in: Int J Pediatr. 2015;2015:235783.
6. Delgado J, Toro R, Rascovsky S, Arango A, Angel GJ, Calvo V, Delgado JA. Chloral hydrate in pediatric magnetic resonance imaging: evaluation of a 10-year sedation experience administered by radiologists. Pediatr Radiol. 2015;45:108-14.
7. Lee YJ, Kim DK, Kwak YH, Kim HB, Park JH, Jung JH. Analysis of the appropriate age and weight for pediatric patient sedation for magnetic resonance imaging. Am J Emerg Med. 2012;30:1189-95.
8. Mason KP, Fontaine PJ, Robinson F, Zgleszewski S. Pediatric sedation in a community hospital-based outpatient MRI center. AJR Am J Roentgenol. 2012;198:448-52.
9. Siddappa R, Riggins J, Kariyanna S, Calkins P, Rotta AT. High-dose dexmedetomidine sedation for pediatric MRI. Paediatr Anaesth. 2011;21:153-8.
10. Litman RS, Soin K, Salam A. Chloral hydrate sedation in term and preterm infants: an analysis of efficacy and complications. Anesth Analg. 2010;110:739-46.
11. Lubisch N, Roskos R, Berkenbosch JW. Dexmedetomidine for procedural sedation in children with autism and other behavior disorders. Pediatr Neurol. 2009;41:88-94.
12. Mason KP, Zurakowski D, Zgleszewski SE, Robson CD, Carrier M, Hickey PR, Dinardo JA. High dose dexmedetomidine as the sole sedative for pediatric MRI. Paediatr Anaesth. 2008;18:403-11.
13. Low E, O'Driscoll M, MacEneaney P, O'Mahony O. Sedation with oral chloral hydrate in children undergoing MRI scanning. Ir Med J. 2008;101:80-2.
14. Phan H, Nahata MC. Clinical uses of dexmedetomidine in pediatric patients. Paediatr Drugs. 2008;10:49-69.
15. Cortellazzi P, Lamperti M, Minati L, Falcone C, Pantaleoni C, Caldiroli D. Sedation of neurologically impaired children undergoing MRI: a sequential approach. Paediatr Anaesth. 2007;17:630-6.
16. Malviya S, Voepel-Lewis T, Tait AR, Reynolds PI, Gujar SK, Gebarski SS, Petter Eldevik O. Pentobarbital vs chloral hydrate for sedation of children undergoing MRI: efficacy and recovery characteristics. Paediatr Anaesth. 2004;14:589-95.

**Supplementary Figure 7. PRISMA flow diagram**

**Footnote: #1:** In addition to studies identified through the primary database search, two eligible articles were identified during reference screening of included meta-analyses. These studies were not captured in the initial search strategy but fulfilled all inclusion criteria and were included in the final analysis.

**From: Page MJ, McKenzie JE, Bossuyt PM, Boutron I, Hoffmann TC, Mulrow CD, et al. The PRISMA 2020 statement: an updated guideline for reporting systematic reviews. BMJ 2021;372:n71. doi: 10.1136/bmj.n71 (For more information, visit:** [**http://www.prisma-statement.org/**](http://www.prisma-statement.org/)**)**

**Supplementary Figure 8-A. Forest plot comparing eligible studies on intravenous midazolam**


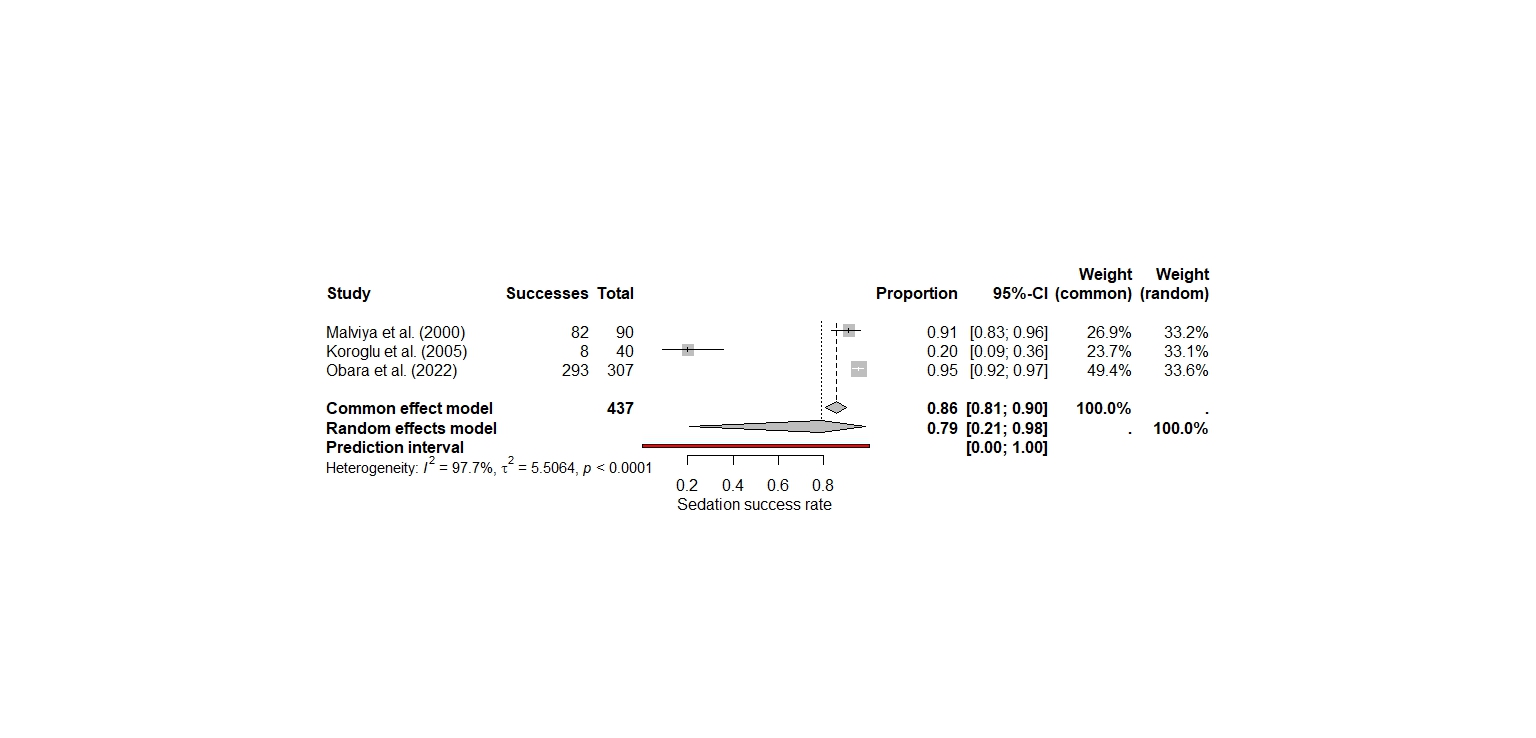


**Supplementary Figure 8-B. Forest plot comparing eligible studies on intravenous propofol**


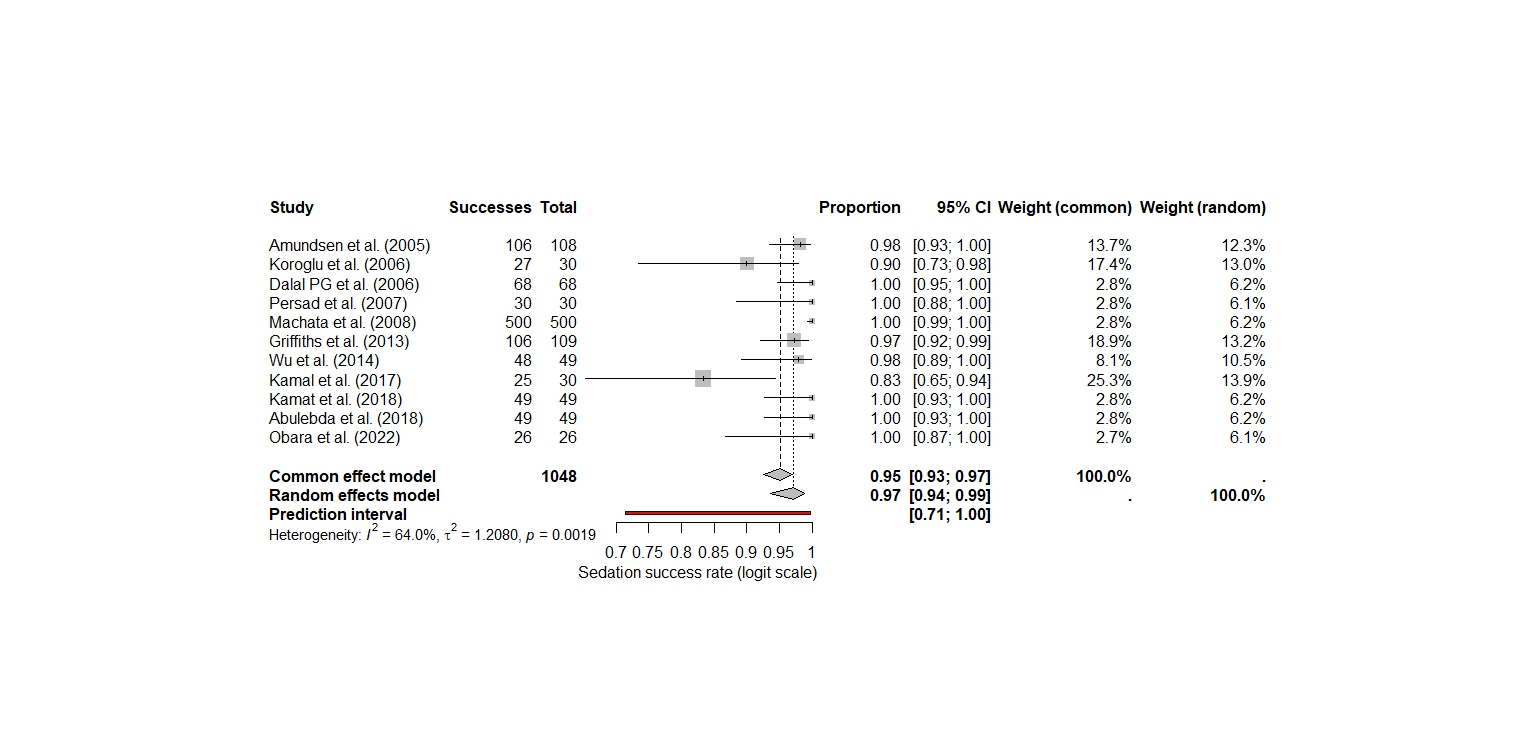


**Supplementary Figure 8-C. Forest plot comparing eligible studies on general anesthesia**


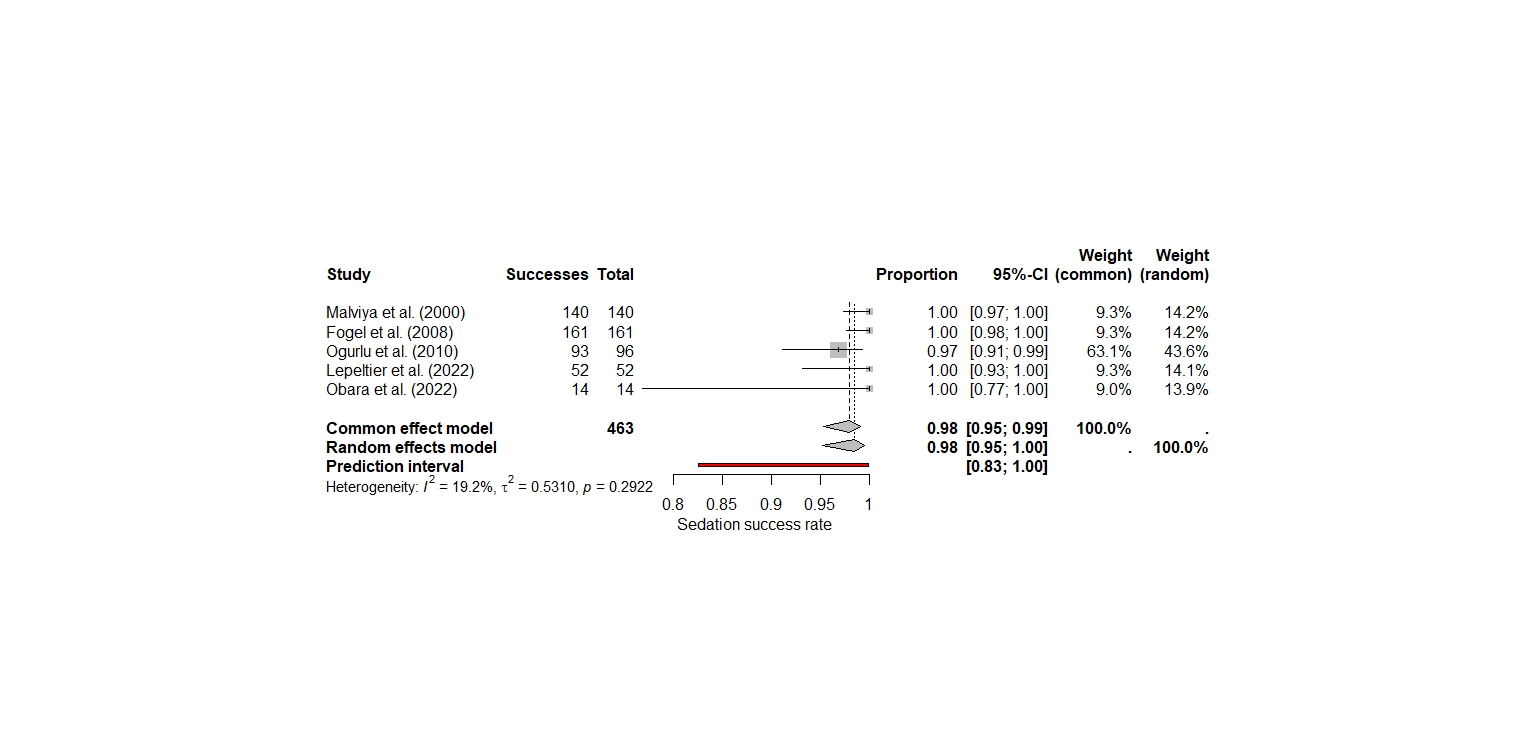


**CHEERS 2022 Checklist**

| **Topic** | **No.** | **Item** | **Location where item is reported** |
| --- | --- | --- | --- |
| **Title** |  |  |  |
|  | 1 | Identify the study as an economic evaluation and specify the interventions being compared. | Title |
| **Abstract** |  |  |  |
|  | 2 | Provide a structured summary that highlights context, key methods, results, and alternative analyses. | Abstract |
| **Introduction** |  |  |  |
| **Background and objectives** | 3 | Give the context for the study, the study question, and its practical relevance for decision making in policy or practice. | Introduction |
| **Methods** |  |  |  |
| **Health economic analysis plan** | 4 | Indicate whether a health economic analysis plan was developed and where available. | Economic Analysis |
| **Study population** | 5 | Describe characteristics of the study population (such as age range, demographics, socioeconomic, or clinical characteristics). | Study Population |
| **Setting and location** | 6 | Provide relevant contextual information that may influence findings. | Setting and Location |
| **Comparators** | 7 | Describe the interventions or strategies being compared and why chosen. | Comparators |
| **Perspective** | 8 | State the perspective(s) adopted by the study and why chosen. | Perspective |
| **Time horizon** | 9 | State the time horizon for the study and why appropriate. | Time Horizon |
| **Discount rate** | 10 | Report the discount rate(s) and reason chosen. | Time Horizon |
| **Selection of outcomes** | 11 | Describe what outcomes were used as the measure(s) of benefit(s) and harm(s). | Outcome (Effectiveness) |
| **Measurement of outcomes** | 12 | Describe how outcomes used to capture benefit(s) and harm(s) were measured. | Outcome (Effectiveness) & Table 1 |
| **Valuation of outcomes** | 13 | Describe the population and methods used to measure and value outcomes. | Outcome (Effectiveness) & Table 1 |
| **Measurement and valuation of resources and costs** | 14 | Describe how costs were valued. | Cost Estimation & Table 1 |
| **Currency, price date, and conversion** | 15 | Report the dates of the estimated resource quantities and unit costs, plus the currency and year of conversion. | Currency and Price Year |
| **Rationale and description of model** | 16 | If modelling is used, describe in detail and why used. Report if the model is publicly available and where it can be accessed. | Model Structure |
| **Analytics and assumptions** | 17 | Describe any methods for analysing or statistically transforming data, any extrapolation methods, and approaches for validating any model used. | Cost Effectiveness Analysis |
| **Characterising heterogeneity** | 18 | Describe any methods used for estimating how the results of the study vary for subgroups. | Not Reported |
| **Characterising distributional effects** | 19 | Describe how impacts are distributed across different individuals or adjustments made to reflect priority populations. | Not Reported |
| **Characterising uncertainty** | 20 | Describe methods to characterise any sources of uncertainty in the analysis. | Cost Effectiveness Analysis |
| **Approach to engagement with patients and others affected by the study** | 21 | Describe any approaches to engage patients or service recipients, the general public, communities, or stakeholders (such as clinicians or payers) in the design of the study. | Not Reported |
| **Results** |  |  |  |
| **Study parameters** | 22 | Report all analytic inputs (such as values, ranges, references) including uncertainty or distributional assumptions. | Table 1 |
| **Summary of main results** | 23 | Report the mean values for the main categories of costs and outcomes of interest and summarise them in the most appropriate overall measure. | Results & Table 2 |
| **Effect of uncertainty** | 24 | Describe how uncertainty about analytic judgments, inputs, or projections affect findings. Report the effect of choice of discount rate and time horizon, if applicable. | One-Way Sensitivity Analysis & Probabilistic Sensitivity Analysis |
| **Effect of engagement with patients and others affected by the study** | 25 | Report on any difference patient/service recipient, general public, community, or stakeholder involvement made to the approach or findings of the study | Not Reported |
| **Discussion** |  |  |  |
| **Study findings, limitations, generalisability, and current knowledge** | 26 | Report key findings, limitations, ethical or equity considerations not captured, and how these could affect patients, policy, or practice. | Discussion |
| **Other relevant information** |  |  |  |
| **Source of funding** | 27 | Describe how the study was funded and any role of the funder in the identification, design, conduct, and reporting of the analysis | Funding |
| **Conflicts of interest** | 28 | Report authors conflicts of interest according to journal or International Committee of Medical Journal Editors requirements. | Declarations |

*From:* Husereau D, Drummond M, Augustovski F, de Bekker-Grob E, Briggs AH, Carswell C, Caulley L, Chaiyakunapruk N, Greenberg D, Loder E, Mauskopf J, Mullins CD, Petrou S, Pwu RF, Staniszewska S; CHEERS 2022 ISPOR Good Research Practices Task Force. Consolidated Health Economic Evaluation Reporting Standards 2022 (CHEERS 2022) statement: updated reporting guidance for health economic evaluations. Value Health. 2022;25:3-9.
